# Increasing Engagement in Kitten Fostering Programs: Lessons Learned From High Kitten Intake Zip Codes in Los Angeles County

**DOI:** 10.3389/fvets.2022.897687

**Published:** 2022-06-09

**Authors:** Shelby E. McDonald, Gregory S. Miller, Tina Reddington Fried, Debra Olmedo, Angela Matijczak

**Affiliations:** ^1^Department of Strategy and Research, American Society for the Prevention of Cruelty to Animals, New York, NY, United States; ^2^American Society for the Prevention of Cruelty to Animals, Feline Programs, Los Angeles, CA, United States; ^3^School of Social Work, Virginia Commonwealth University, Richmond, VA, United States

**Keywords:** kittens, fostering, Latinx, animal welfare, community engagement, shelter intake, diversity & inclusion, community cats

## Abstract

The goal of the current study was to identify ways to increase awareness and engagement in kitten fostering programs (KFPs) among residents of areas with a high intake of kittens to animal shelters in Southern California (i.e., Los Angeles County). Specifically, we aimed to understand residents': (1) awareness of KFPs and kitten overpopulation issues, (2) interest in fostering kittens with an animal welfare organization, (3) concerns about fostering, (4) perceived ability to meet common KFP requirements, and (5) perceptions of potential KFP marketing/messaging and communication methods. Participants included 283, predominantly Hispanic/Latinx adults aged 18 years or older who resided in Los Angeles County and who lived in one of 12 zip codes with a high rate of kitten shelter intake. Survey results indicated that more than one quarter of participants had engaged in fostering on their own without an animal shelter or rescue program. One-third of the total sample, and more than two-thirds of participants who had already fostered cats and kittens on their own, were open to fostering kittens in partnership with an animal shelter. A majority of individuals who were interested in fostering had not seen advertising for fostering programs; Spanish-language participants were significantly less likely than expected to have encountered program advertisements. The most prevalent concerns about fostering in our sample were centered on the time (79%), cost (78%), and space (77%) required to engage in fostering. Text, email, social media, and mail were among the most preferred methods for marketing and communication, with some variation between Spanish and English language respondents. Opportunities for increasing engagement included, but were not limited to, improving the promotion of program advertisements using animal-welfare and cost-focused messaging approaches and improving the dissemination and marketing of Spanish-language materials. Providing community members with realistic expectations of the time, resources, and support they will get from animal welfare organizations may improve engagement in KFPs, as well as identifying alternative resources and supports (e.g., transportation, in-home veterinary visits) to assist community members in serving animals in their community.

## Introduction

Fostering programs that place kittens (and other animals) temporarily in volunteers' homes play a critical role in improving animal welfare and preventing and reducing shelter overcrowding and euthanasia (1). It has therefore become increasingly common for kittens in the care of animal welfare organizations to be reared in foster care until they are considered ready for adoption (2). This method of care has gained increased attention throughout the COVID-19 pandemic, during which public health protocols required rapid adaptations to standard shelter operations and led many organizations to evaluate the benefits, viability, and animal welfare outcomes associated with volunteer fostering programs (3).

Foster care-based kitten rearing programs help to reduce shelter overcrowding and improve animal welfare organizations' standard of care for kittens. Among animals that enter shelters, kittens face particularly high risk for mortality with rates ranging from 15 to 40% across studies (4, 5). Foster care programs help to mitigate concerns regarding disease exposure and transmission, inadequate nutrition, and stress (6). Along with these notable impacts on animal welfare, it is hypothesized that foster care-based rearing may have positive impacts on kittens' long-term behavioral development by providing increased enrichment and early socialization. Early socialization plays a significant role in the development of adult cat behaviors, including aggression and fear (2). Thus, the potential for increased socialization and reduced stress associated with foster environments may be particularly important for reducing aggressive behaviors that can harm the owner-cat relationship and contribute to relinquishment or abandonment of the animal and/or euthanasia pre- and post-adoption (7, 8).

### Encouraging Community Care

Given the benefits of fostering programs to animal welfare and organizational capacity, there is a growing interest in identifying strategies and best practices for engaging communities in foster care programs (9–11). A 2015 report by Maddie's Fund (10) indicated that 78% of animal welfare organizations surveyed (i.e., U.S. rescue organizations, municipal animal control agencies, and animal shelters) were likely or extremely likely to encourage community members who found kittens to care for them when organizational resources were not available or until kittens were ready to be placed for adoption, with highest rates reported among shelters without government contracts (85%). Among organizations that encouraged community foster care of kittens, 43% reported that few or very few community members elected to provide care for kittens, 41% endorsed that “some” elected to provide care, and only 16% endorsed that most or many elected to provide care.

A later survey conducted by Maddie's Fund (11) aimed to identify a target audience of prospective foster caregivers, barriers they face to fostering, and the best communication methods to engage them. This study found that prospective foster caregivers (*N* = 1,079) most often heard about fostering through someone they knew who had fostered (35%); only 19% of prospective fosters reported hearing about fostering through a rescue organization or shelter, whereas almost half (46%) of active foster caregivers heard about fostering through a rescue organization or shelter. Among prospects, the primary reasons for being hesitant to foster included becoming too attached to a foster animal (25%), not having enough time to care for them (24%) and having other pets in the household (18%). Those who were most interested in fostering were more likely to be younger, live alone, and demonstrate more awareness of animal homelessness. Maddie's Fund also reported that participants endorsed social media most frequently as the most effective way to bring attention to fostering needs, followed by news stories about fostering. Another notable finding of the 2017 Maddie's Fund report was that most prospective fosters showed an interest in fostering adult dogs (36%) and senior pets (35%), with only 22% and 16% of participants expressing interest in cats and kittens, respectively. Although replication is needed, these reports by Maddie's Fund (10, 11) provide preliminary evidence that identifying strategies to recruit, engage, and retain caregivers for kitten fostering programs (KFPs) should be an important priority for animal welfare organizations. To our knowledge, no published studies or reports have specifically sought to identify barriers and opportunities to increase kitten fostering. The current study makes an important contribution to the literature by addressing this gap in research.

### High-Kitten Intake Communities

There has been increasing interest in the intersection between animal intake and human vulnerability in communities, as well as promoting equity in animal sheltering services and ensuring animal welfare organizations are serving all members of their communities (12). For example, Best Friends Animal Society released a report indicating that high vulnerability counties within the United States (e.g., low socioeconomic status, racially minoritized populations) have a higher rate of animal intake overall compared to nation-level rates and that rates of adoptions, as a proportion of intake, were lower in areas with high levels of human social vulnerability (13). Relatedly, a recent study in Canada reported that the situational vulnerability (e.g., income, education) of communities to which kittens were adopted was significantly lower (less vulnerable) than the vulnerability of communities where kittens originated, indicating the flow of kittens from more vulnerable to less vulnerable communities (14). Although most work in this area has focused on the role of owner surrender and adoption, the role of fostering programs also warrants attention as a potential way for shelters to identify inequities and to intervene to reduce intake and increase adoptions in high-intake communities, as well as improve the sustainability of fostering programs. We are unaware of any published studies that have sought to identify ways to increase the engagement of community members in high-kitten intake neighborhoods in local fostering programs.

### Current Study

The overarching goal of this cross-sectional study was to identify barriers and opportunities to increase KFP volunteers among residents of twelve Los Angeles County zip codes with a high intake of kittens to animal shelters. Specifically, we aimed to better understand five specific areas related to fostering in high kitten shelter-intake (HKSI) areas: 1) community members' awareness of KFPs and kitten overpopulation issues, 2) community members' interest in fostering kittens with an animal welfare organization, 3) community members' worries about fostering, 4) community members' ability to meet common KFP requirements, and 5) community members' perceptions of potential marketing/messaging and communication methods. We also conducted exploratory bivariate analyses to understand whether the sociodemographic characteristics of community members were associated with their responses to questions in each of these domains.

## Method

### Study Design

**Figure 1:**
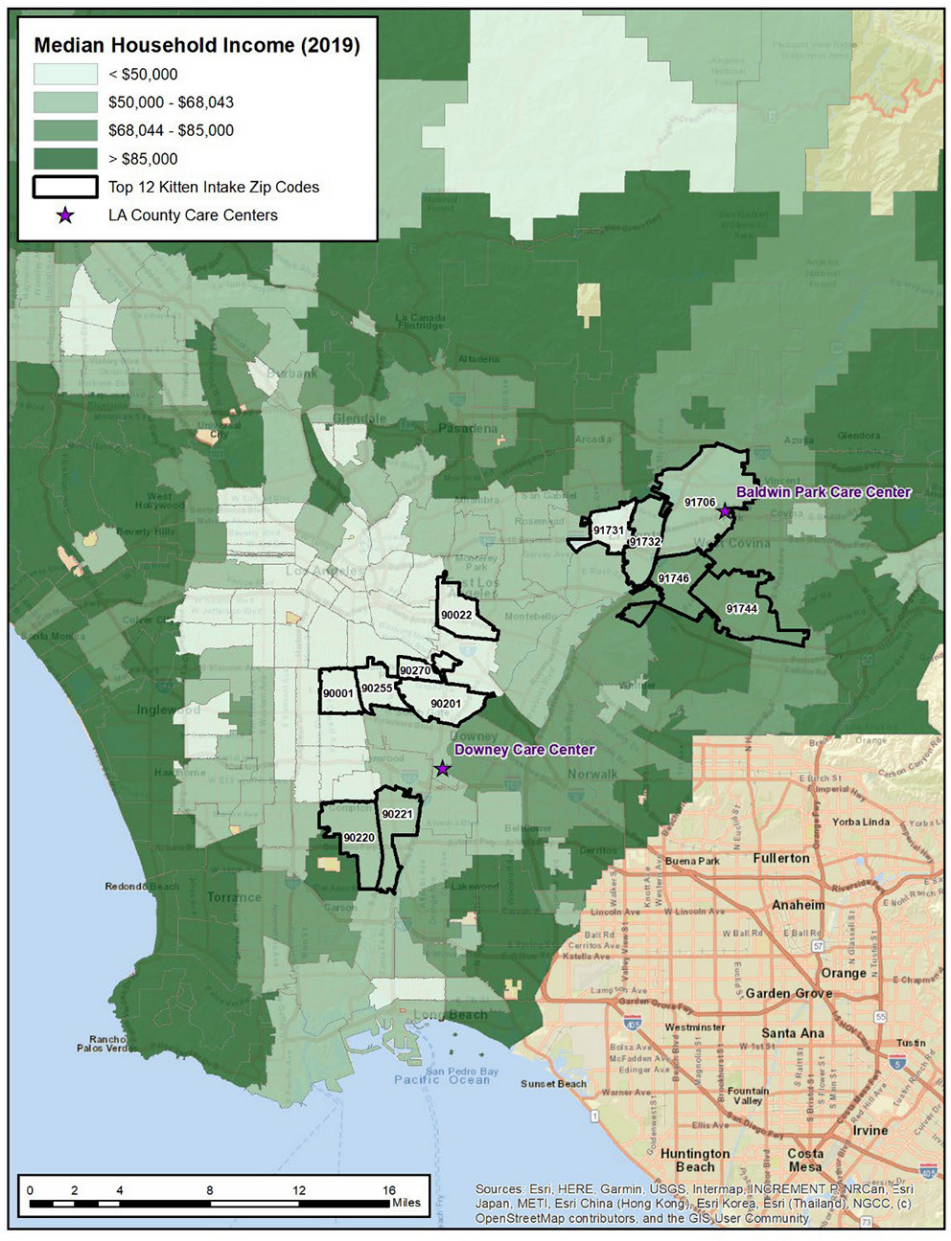
Median household income.

We conducted a cross-sectional survey of current and former clients of the American Society for the Prevention of Cruelty to Animals (ASPCA) who had received some service or assistance (e.g., pet medical care, pet food, etc.) from the ASPCA from January 2019 to October 2020. Data for the current study were collected between July 12, 2021, and August 31, 2021. Participants included 283 adults aged 18 years or older who resided in California and who lived in 12 zip codes with a high rate of kitten shelter intake. All procedures were approved by Advarra IRB (Protocol Number: Pro00050534). Our rationale for this convenience sampling strategy was 2-fold: (1) we hypothesized that community members who had previously reached out to assist animals would, potentially, have a higher likelihood of becoming a foster than the general population, and (2) their contact information was readily available to the organization. With shelter intake data provided by the LA County Department of Care and Control (DACC), we identified HKSI zip codes within the Baldwin Park and Downey Animal Care Center service areas. Specifically, we examined data collected between January 2019 and October 2020 and summarized kitten (cats <5 months old) intake counts by zip code and divided this number by the estimated number of households per zip code (total kitten intake/estimated number of households) to determine the top 12 zip codes for inclusion (>0.040 kitten intakes per household). Figure 1 provides a visual of the HKSI zip codes and care center locations.

After determining the 12 target zip codes, we identified ASPCA contacts from the organization's client management system that had addresses within any of these zip codes. These clients had engaged with the organization for medical services (Spay/Neuter Services, Primary Pet Care, a subsidy program for veterinary care) and/or food distributions (received free pet food) and had received services between January 2019 and November 2020. Specifically, we randomly selected 50% of our total contacts from each target zip code, which yielded a pool of 3,719 potential participants. Participants were recruited via text messages that read “Hello from the ASPCA! We would like to hear from you regarding kittens in your community. Can you complete this 5–10-min survey? As a thank you for your time, we will randomly select five survey respondents to receive a $50 Amazon gift card.” This message was available in English and Spanish language versions and included a direct link to the survey. Participants who did not complete the survey following the first contact received one additional message which included text similar to the first contact. The median completion time was ~9 min. Answers were captured electronically using SurveyMonkey.

### Participants

Collectively, the 12 zip codes targeted in our study had a total population of 717,170 and included 173,838 households (15). US Census American Community Survey estimates, accessed via Esri ArcGIS Community Analyst (16), indicated that the median household income was $51,488 and 20% of households reported annual income below the poverty level. In comparison, Los Angeles County median household income was $68,044 with 15% of households reporting annual income below the poverty level. Eighty-three percent of the study area population identified as Hispanic or Latinx (compared to 49% for LA County). In terms of educational attainment, 6% of study area adults aged 25 years or older had no formal schooling compared to 4% for LA County. Approximately 41% of study area adults aged 25 years or older had less than a high school diploma/GED (21% for LA County) and 14% had an associate degree or higher education (40% for LA County).

#### Subsample Characteristics

Fifty-six percent (*n* = 159) of individuals who started the survey completed all survey questions including the demographic questions that concluded the survey. The subsample that completed all survey questions predominantly identified as women (77%), Hispanic/Latinx (78%), had an annual household income under $50K (66%), and currently had at least one pet (97%). Seventy-four percent completed the English-language version of the survey. Language preference for completing the survey was not significantly associated with the likelihood of completing all survey questions. Sociodemographic details for the subsample are included in Table 1.

**Table 1:** Demographic information of respondents.

**Variable name**	**Variable categories**	***n***	**%**
Racial/ethnic identity (*n* = 157)	African American or Black	5	3.2
	Asian	1	0.6
	Hispanic/Latinx	122	77.7
	Middle Eastern	4	2.5
	Native American or Alaska Native, Native Hawaiian or other Pacific Islander	1	0.6
	White	7	4.5
	Prefer not to answer	11	7.0
	Multiple selected	6	3.8
Gender identity (*n* = 157)	Man	24	15.3
	Woman	122	77.7
	Gender minority	3	1.9
	Prefer not to answer	8	5.1
Language spoken at home (*n* = 157)	Chinese	1	0.6
	English	61	38.9
	Spanish	38	24.2
	English and Spanish	55	35.0
	Prefer not to answer	2	1.3
Age (*n* = 158)	18–24	17	10.8
	25–34	31	19.6
	35–44	40	25.3
	45–54	28	17.7
	55–64	27	17.1
	65+	11	7.0
	Prefer not to answer	4	2.5
Household income (*n* = 153)	$0–$25,000	51	33.3
	$25,001–$50,000	50	32.7
	$50,001–$75,000	16	10.5
	$75,001–$100,000	6	3.9
	$100,001–$125,000	1	0.7
	$125,001–$150,000	2	1.3
	>$150,000	0	0.0
	Prefer not to answer	28	9.9

### Survey Content

The survey included 36 questions spanning several content areas. For the purpose of the survey, we provided participants with a definition of fostering, which read: “fostering kittens refers to temporarily caring for orphaned kittens in your home who are not yet ready for adoption.” Community members' awareness of KFPs and kitten overpopulation issues was assessed using a total of three questions. The first question asked participants to indicate whether they had seen any advertising or promotions for KFPs, using the response options of yes, no, or unsure. An additional two questions asked respondents to indicate the most common actions taken for outdoor adult cats in their neighborhood and outdoor kittens in their neighborhood. Both of these questions offered several response options (e.g., “I take care of them,” “They are brought to the shelter”), as well as an option to write in their own response. Participants who selected “unsure” were grouped with those who had not encountered KFPs for the purposes of our analyses.

To assess community members' interest in fostering kittens, we utilized questions asking participants to indicate their interest in three fostering scenarios: (1) a friendly adult mother cat and her kittens, (2) up to two orphaned kittens at the same time, and (3) up to five orphaned kittens at the same time. Participants were able to indicate their interest in each situation using a 5-point Likert scale that ranged from “not interested at all” to “very interested.” Participants were provided with an image illustrating each scenario to ensure all participants had comparable references for adult cats and kittens. Each question was analyzed separately in order to obtain nuanced information based on various fostering scenarios.

Community members' potential concerns about fostering were assessed using 11 questions in which participants indicated how worried they were regarding specific fostering situations (e.g., “It will take too much time on a daily basis,” “The kittens will make my pets sick”). Response options for these questions ranged from 0 (not worried at all) to 5 (extremely worried). Each item was analyzed separately with the goal of understanding malleable targets for reducing barriers to participation in KFPs.

Community members' ability to meet common KFP requirements was measured using a total of 10 questions across five domains of requirements: time commitment (two questions; e.g., “Please rate how able you are to foster kittens in your own home for 1–6 days”), transportation [two questions; e.g., “Please rate how able you are to transport your foster kittens immediately (24/7) to a veterinary clinic in case of emergency”], home environment (one item; “Please rate how able you are to set up an ~4-foot wide playpen provided by the ASPCA for your foster kittens to stay inside in your home and keep them separated from your own pets”), communication (two questions; e.g., “Please rate how able you are to attend an online fostering training session for 1 h”), and daily care (three questions; e.g., “Please rate how able you are to bottle feed foster kittens every 2–5 h”). All questions used a 4-point Likert scale that ranged from 1 (never able to meet this responsibility) to 4 (always able to meet this responsibility). In addition to examining each question, we created a total score that reflected the number of common KFP requirements that respondents could provide (McDonald's ω = 0.81).

We assessed community members' perceptions of potential marketing/messaging and communication methods using two questions. The first item asked participants to indicate what type of message would most encourage them to join a kitten foster program; response options included messages that discussed the ways that fostering benefitted the health of kittens, the ability of the ASPCA to provide financial support and resources, the ability of the ASPCA to provide additional support (e.g., medical helpline), that fostering was easier than expected, and a quotation from a current foster reflecting their positive experience. The second question asked participants to select the three best ways of sharing information regarding KFPs in their area. Respondents were able to choose from a variety of responses (e.g., mail, email, TV) and write in their own response, if needed.

Finally, the survey included six demographic questions to collect data on the participants' race/ethnicity (categorical), gender (categorical), age (categorical), annual household income (categorical), primary language (categorical), and current pet ownership (categorical).

Surveys were available in English and Spanish language versions. We partnered with a professional translation company (Language Line) to translate the English version of the survey to Spanish. In addition, bilingual ASPCA staff who worked within our target communities provided feedback on the surveys following translation and made minor adjustments to ensure the appropriateness of the survey for local Spanish-speaking study participants.

### Analysis

All descriptive and bivariate analyses were conducted in SPSS version 28. Percentages reported are valid percentages (count divided by the total number of valid, non-missing observations). We conducted a series of Pearson chi-square tests of proportion to examine associations between our constructs of interest (prior foster experience, interest in fostering, concerns about fostering, and ability to meet common fostering program requirements). We also conducted a series of independent sample *t*-tests to determine whether the average number of common program requirements that could be met was significantly different between those who were or were not interested in our three fostering scenarios. All test assumptions were tested and met.

#### Subsample Analysis

Using data from the subsample of participants who had complete data for all survey questions, we conducted a series of Pearson chi-square tests to examine whether sociodemographic characteristics (age, Latinx/Hispanic ethnicity, gender, and income level) were associated with awareness of KFPs, fostering experience and interest, actions participants would take for outdoor cats in their community, and their ability to meet foster program requirements. We also examined whether sociodemographic characteristics were associated with participants' preference for potential marketing/messaging and communication methods. Prior to analysis the demographic variables were recoded to avoid empty cells and small cell sizes. Age was recoded into three groups: 18–34, 35–54, and 55 years and older. Gender was recoded so that participants who identified with a gender minority identity (e.g., agender, non-binary) were grouped with participants who identified as women; the rationale for this decision was to avoid dropping gender minority participants from the sample and to combine participants who experience gender-related inequality into one category (17). All other racial/ethnic identities were compared to Hispanic/Latinx participants. Finally, income was recoded into three categories: $0–$25,000, $25,001–$50,000, $50,001 or more.

When a statistically significant chi-square test was found using a standard alpha threshold of *p* = 0.05, we conducted *post-hoc* analyses of the standardized adjusted residuals for each cell in each contingency table to interpret the significant difference (18). Critical thresholds for these analyses were Bonferroni corrected to reduce the risk of Type I error.

## Results

### Fostering Experience, Program Awareness, and Knowledge of Kitten Overpopulation Issues

Survey questions and endorsement rates for fostering experience, program awareness, and knowledge of kitten overpopulation issues are provided in Table 2. Chi-square tests of independence indicated that age, gender, Latinx/Hispanic ethnicity, and income were not significantly associated with participants' exposure to KFP promotion or advertisement materials. Participants' preferred language for completing the survey (Spanish or English) was significantly associated with having encountered KFP advertisements or promotional materials. Spanish-language respondents were less likely than expected (5 vs. 18%; adjusted residual = −2.7) to have seen promotional materials or advertisements and English language participants were more likely than expected (23 vs. 19%; adjusted residual = 2.7) to have encountered these materials, $X_{\left(2,155\right)}^{2}$ = 6.16, *p* = 0.03, Cramer's *V* = 0.738. Sociodemographic characteristics were not significantly associated with prior foster experience. Due to small cell sizes, we were unable to examine associations between participants' sociodemographic characteristics and perceptions of actions taken to care for community cats.

**Table 2:** Fostering experience, program awareness, and knowledge of kitten overpopulation issues.

**Question**	**Response categories**	***n***	**%**
Please select the statement that best reflects your experience with kitten fostering. You may select all statements that apply (*n* = 283).	I have never fostered kittens and no one I know has ever fostered kittens.	147	51.9
	I have fostered kittens on my own without an animal shelter or rescue foster program.	73	25.8
	Someone I know has fostered kittens (with an animal shelter/rescue or on their own).	58	20.5
	I have fostered kittens with an animal shelter or rescue foster program.	8	2.8
Have you ever seen any advertising or promotions for a kitten	No	194	68.6
fostering program? (*n* = 283)	Yes	55	19.4
	Unsure	34	12.0
When thinking about outdoor cats in your neighborhood, what do you think is the most common action taken for them? (*n* = 252)	I take care of them, and other people take care of them	53	21.0
	I take care of them	52	20.6
	No one is taking care of them	49	19.4
	Other people take care of them	42	16.7
	Not sure	34	13.5
	N/A: There are 0 outdoor adult cats in my neighborhood	13	5.2
	They are brought to the shelter	5	2.0
	Other	4	1.6
When thinking about outdoor kittens in your neighborhood, what do	No one is taking care of them	49	19.7
you think is the most common action taken for them? (*n* = 249)	I take care of them, and other people take care of them	46	18.5
	I take care of them	44	17.7
	Other people take care of them	33	13.3
	Not sure	33	13.3
	N/A: There are 0 outdoor kittens in my neighborhood	25	10.0
	They are brought to the shelter	14	5.6
	Other	5	2.0

### Interest in Providing Foster Care for Cats and Kittens in Their Community

A summary of responses pertaining to respondents' interest in fostering is provided in Table 3. To assess relationships between participants' demographic characteristics and interest in fostering, response categories for the interest in fostering variables were collapsed to avoid small/empty cell sizes. Responses of “not interested at all” and “not interested” were collapsed into one category indicating a lack of interest in fostering (0) whereas responses that indicated being neutral to, interested, or very interested in fostering were collapsed into another category that reflected openness to fostering (1). More than 50% of individuals who were open to fostering had not seen any advertising or promotional materials for KFPs. Thirty-four percent of participants were open to all three fostering scenarios (φ_range_ = 0.71–0.83). Age, gender, Latinx/Hispanic ethnicity, and income level were not significantly associated with interest in fostering, and this was consistent across all three fostering scenarios (adult cat with kittens, 2 kittens, 5 kittens). Participants' language preference for completing the survey was not significantly associated with interest in fostering an adult cat with kittens. However, we found significant associations between language preference and openness to fostering up to two and five kittens. Specifically, participants who responded in Spanish were significantly less likely than expected to be open to fostering up to two kittens (28 vs. 47%; adjusted residual = −2.6), whereas English-language respondents were more likely than expected (53 vs. 47%; adjusted residual = 2.6) to be interested in fostering up to two kittens $X_{\left(1,150\right)}^{2}$ = 6.79, *p* = 0.009, Cramer's *V* = 0.213. Similar results were found for fostering up to five kittens, with Spanish-language participants being less likely than expected to be open to fostering (25 vs. 40%; adjusted residual = −2.1) and English language respondents being more likely than expected (45 vs. 40% standardized adjusted residual = 2.1) to be open to fostering, $X_{\left(1,150\right)}^{2}$ = 4.44, *p* = 0.035, Cramer's *V* = 0.172.

**Table 3:** Interest in providing foster care for cats and kittens.

**How interested are you in fostering kittens in the following situations?**	**Not interested at all *n* (%)**	**Not interested *n* (%)**	**Neither not interested or interested *n* (%)**	**Interested *n* (%)**	**Very interested *n* (%)**
Fostering up to 5 orphaned kittens at the same time (*n* = 260)	95 (36.5)	87 (33.5)	48 (18.5)	19 (7.3)	11 (4.2)
Fostering a friendly adult mom cat and her kittens (*n* = 263)	79 (30.0)	81 (30.8)	58 (22.1)	31 (11.8)	14 (5.3)
Fostering up to 2 orphaned kittens at the same time (*n* = 259)	79 (30.5)	85 (32.8)	44 (17.0)	35 (13.5)	16 (6.2)

Next, we examined whether individuals who had fostered on their own were more or less likely to be interested in fostering with an animal welfare organization using a series of chi-square tests. Participants who had previously fostered on their own without an animal welfare organization were more likely than expected (69 vs. 48%) to be interested in fostering an adult cat and kittens in partnership with an animal welfare organization, $X_{\left(1,156\right)}^{2}$ = 10.973, *p* < 0.001, Cramer's *V* = 0.265. Similar results were found for fostering up to two kittens (observed = 72%; expected = 46%, $X_{\left(1,154\right)}^{2}$ = 16.21, *p* < 0.001, Cramer's *V* = 0.325) and up to five kittens (observed = 63% expected = 43%, $X_{\left(1,154\right)}^{2}$ = 14.25, *p* < 0.001, Cramer's *V* = 0.325).

### Ability to Meet Common Foster Program Requirements

Questions relating to common KFP requirements and endorsement rates for each response category are listed in Table 4. To avoid small cell sizes, the program requirement variables were transformed so that the 4-point rating scale was reduced to a dichotomous scale; the response categories of never and rarely were combined to indicate those who could not consistently meet program requirements (0), while sometimes and always were collapsed into one category reflecting those who could typically meet program requirements. Gender, Latinx/Hispanic ethnicity, and income were not significantly associated with participants' ability to meet common foster program requirements. Age was associated with one program requirement: participants' ability to promote adoptable kittens to friends and family, $X_{\left(2,150\right)}^{2}$ = 6.80, *p* = 0.034, Cramer's *V* = 0.21. *Post-hoc* analyses of the standardized adjusted residuals indicated that participants over 55 years of age were less likely to be able to promote pets to friends and family compared to other age groups (20 vs. 24%; adjusted residual = −1.6), whereas those who were between 18 and 34 years were more likely than expected to say they could regularly promote adoptable kittens to friends and family (40 vs. 32%; adjusted residual = 2.5).

**Table 4:** Ability to meet common foster program requirements.

**Please rate how able to you are (given your current circumstances) to meet the following responsibilities**	**Never able *n* (%)**	**Rarely able *n* (%)**	**Sometimes able *n* (%)**	**Always able *n* (%)**
Foster kittens in your home for 1 week to 1 month (*n* = 231)	124 (53.7)	10 (17.3)	46 (19.9)	21 (9.1)
Foster kittens in your home for 1–6 days (*n* = 237)	120 (50.6)	35 (14.8)	54 (22.8)	28 (11.8)
Transport your foster kittens immediately (24/7) to a veterinary clinic in case of emergency (*n* = 228)	116 (50.9)	41 (18.0)	29 (12.7)	42 (18.4)
Set up an approximately 4-ft wide playpen provided by the ASPCA for your foster kittens to stay inside in your home and keep them separated from your own pets (*n* = 219)	115 (52.5)	29 (13.2)	36 (16.4)	39 (17.8)
Transport your foster kittens to scheduled medical appointments <10 miles from your home (*n* = 233)	114 (48.9)	44 (18.9)	44 (18.9)	31 (13.3)
Attend an online kitten foster training session for 1 h (*n* = 211)	103 (48.8)	27 (12.8)	47 (22.3)	34 (16.1)
Bottle feed foster kittens every 2–5 h (*n* = 194)	96 (49.5)	28 (14.4)	39 (20.1)	31 (16.0)
Hand feed foster kittens up to 6 times a day (*n* = 191)	95 (49.7)	30 (15.7)	39 (20.4)	27 (14.1)
Give plated food to foster kittens up to 4 times a day (*n* = 193)	88 (45.6)	18 (9.3)	46 (23.8)	41 (21.2)
Promote adoptable kittens to your friends, family and neighbors (*n* = 200)	63 (31.5)	32 (16.0)	58 (29.0)	47 (23.5)

We also tested whether participants' interest in fostering was associated with their ability to meet common KFP requirements. Chi-square tests of independence indicated that openness to fostering was significantly associated with participants' ability to meet common foster program requirements, and this finding was consistent for all 10 requirements examined in the current study (Cramer's V range = 0.37–0.62; *p* < 0.001); moreover, this association was evident for those open to fostering up to two kittens, up to five kittens, and/or an adult cat with kittens. Specifically, participants who indicated an openness to fostering were more likely than expected to be able to meet each of the fostering program requirements examined in this study (56–79%; adjusted residuals range = 4.0–7.1).

To understand whether there were significant differences between those who were and were not interested in fostering regarding the number of program requirements that could be met, three exploratory independent sample *t*-tests were performed. Specifically, we examined whether the mean number of program requirements that could be met differed between participants who were and were not open to fostering. Regardless of the fostering scenario (adult cat with kittens, 2 kittens, 5 kittens), participants who were interested in fostering could, on average, meet between five and seven program requirements, whereas those who were not open to fostering could meet one or two out of 10 (*p* < 0.001). Results of the *t*-test analyses are provided in Table 5.

**Table 5:** Relationship between fostering interest and number of program requirements able to be met.

**Fostering interest**		***n***	***M***	***SD***	***t***	**95% Confidence intervals**	
						***LL***	***UL***
Adult cat with kittens	Yes	96	5.86	3.75	−10.18^***^	−5.27	−3.56
	No	142	1.45	2.43			
2 kittens	Yes	89	6.19	3.62	−10.64^***^	−5.53	−3.80
	No	145	1.52	2.56			
5 kittens	Yes	73	6.10	3.70	−8.21^***^	−5.04	−3.08
	No	162	2.04	3.03			

^***^p < 0.001.

*M, mean; SD, Standard Deviation; LL, lower limit; UL, Upper Limit*.

### Concerns About Fostering

Table 6 provides each item relating to worries about fostering and the endorsement rate for each response category. We examined associations between sociodemographic characteristics and fostering-related worries using a series of chi-square tests of independence. Due to small cell sizes, the 5-point rating scale used to assess participants' level of worry was recoded to produce a 3-point scale such that the response categories of “not at all worried” and “slightly worried” were collapsed into one category (0), “somewhat worried” was retained as a category (1), and “moderately” and “extremely worried” were collapsed into a third category that reflected the highest level of worry (2). Participants' age, gender, Latinx/Hispanic ethnicity, and income were not significantly associated with worries about fostering. However, participants' language preference was significantly related to concerns about two aspects of fostering: attachment to kittens and time. Specifically, Spanish-language participants were less likely than expected to have high levels of worry regarding attachment (18 vs. 32%; adjusted residual = −2.1), whereas participants who completed the survey in English were more likely than expected to have high levels of worry (37 vs. 32%; adjusted residual = 2.1), $X_{\left(2,154\right)}^{2}$ = 8.21, *p* = 0.016, Cramer's *V* = 0.23. Similarly, Spanish-language participants were less likely than expected (18 vs. 32%; adjusted residual = −2.1) to have high levels of worry about fostering taking too much time on a daily basis and English language participants were more likely than expected (37 vs. 32%; adjusted residual = 2.1) to have a high level of worry about the time commitment, $X_{\left(2,151\right)}^{2}$ = 7.17, *p* = 0.028, Cramer's *V* = 0.23.

**Table 6:** Concerns about fostering.

**When considering fostering kittens in your home, how worried are you about the following topics**	**Extremely worried** ***n* (%)**	**Moderately worried** ***n* (%)**	**Somewhat worried** ***n* (%)**	**Slightly worried** ***n* (%)**	**Not worried at all** ***n* (%)**
I don't have enough space in my home (*n* = 177)	85 (48.0)	19 (10.7)	19 (10.7)	14 (7.9)	40 (22.6)
I am not home enough (*n* = 177)	78 (44.1)	18 (10.2)	27 (15.3)	14 (7.9)	40 (22.6)
My current pets will not get along with the kittens (*n* = 177)	73 (41.2)	26 (14.7)	24 (13.6)	13 (7.3)	41 (23.2)
It will cost too much money (*n* = 176)	64 (36.4)	33 (18.8)	25 (14.2)	15 (8.5)	39 (22.2)
I will have the kittens in my home for too long before they are adopted (*n* = 177)	60 (33.9)	32 (18.1)	25 (14.1)	18 (10.2)	42 (23.7)
I will become too attached to the kittens to give them back (*n* = 179)	57 (31.8)	23 (12.8)	31 (17.3)	12 (6.7)	56 (31.3)
It will take too much time on a daily basis (*n* = 176)	53 (30.1)	29 (16.5)	36 (20.5)	21 (11.9)	37 (21.0)
I will not receive enough support from the foster program (*n* = 175)	50 (28.6)	19 (10.9)	36 (20.6)	17 (9.7)	53 (30.3)
I don't have enough experience (*n* = 177)	49 (27.7)	17 (9.6)	28 (15.8)	22 (12.4)	61 (34.5)
The kittens will make me or those who live with me sick (*n* = 176)	37 (21.0)	13 (7.4)	15 (8.5)	22 (12.5)	89 (50.6)
The kittens will make my pets sick (*n* = 176)	36 (20.5)	14 (8.0)	23 (13.1)	22 (12.5)	81 (46.0)

### Perception of Potential Marketing/Messaging and Preferred Communication Methods

Marketing messages and endorsement rates are provided in Table 7. Chi-square tests of independence were run to examine associations between sociodemographic characteristics and participants' messaging preferences. Due to small cell sizes, we only examined forms of messaging that were endorsed by at least 20% of the sample. Participants who completed the survey in Spanish were less likely than expected (20 vs. 38%, adjusted residual = −2.6) to endorse social media among the top 3 ways to communicate about KFPs in contrast to English-language participants who were more likely than expected to endorse social media as a top 3 communication strategy (44 vs. 38%, adjusted residual = 2.6), $X_{\left(1,155\right)}^{2}$ = 7.46, *p* = 0.016, Cramer's *V* = 0.219. Text, email, and mail were the top three choices among Spanish-language respondents, whereas text, email and social media were ranked as the top three among English-language respondents. Age was also associated with selecting social media as a top 3 communication strategy, $X_{\left(2,155\right)}^{2}$ = 8.50, *p* = 0.014, Cramer's *V* = 0.235. Participants in the 18–25 years old range were more likely than expected to endorse social media (52.1 vs. 38.3%; adjusted residual = 2.4), whereas participants 55 years of age or older were less likely than expected to endorse social media (22 vs. 37%; adjusted residual = −2.5). No other statistically significant associations were found.

**Table 7:** Perception of potential marketing/messaging and preferred communication methods.

**Topic/question**	**Response categories**	***n***	**%**
Which of the following messages would most encourage you to join a kitten foster program? (*n* = 140)	Most kittens are too young to survive in animal shelters. They are likely to become ill and may remain fearful of people. The ASPCA needs caring people like you to foster orphaned kittens until they are ready to be adopted by loving families.	56	40.0
	When you foster kittens with us, all medical care and supply costs are covered and provided by the ASPCA.	33	23.6
	You are never alone when fostering kittens with the ASPCA. We have a 24/7 medical helpline for any questions or help that you need.	29	20.7
	Fostering kittens is much easier than you think, and you can have a full-time job while doing it! All the kittens need from you is food, playtime, and a little TLC.	15	10.7
	“Fostering has brought me so much fun and joy in my life. I wouldn't trade it for anything!”—ASPCA Foster Erin	7	5.0
Select the 3 best ways for an organization to	Email	64	40.8
share information with you on kitten foster	Texts	64	40.8
programs available in your area? (*n* = 157)	Social media	63	40.1
	Mail	51	32.5
	Pet stores and veterinary clinics	36	22.9
	TV	34	21.7
	Community events	29	18.5
	Shelter/animal care center	22	14.0
	Local schools	21	13.4
	Friends and family	17	10.8
	Newspaper	9	5.7
	Nextdoor app	8	5.1
	Celebrity advertisement	7	4.5

## Discussion

To our knowledge, this is the first study to report on awareness of KFPs in HKSI zip codes and to identify barriers to and opportunities for proactively engaging community members from communities in Los Angeles County. Our study of 283 adults indicated that most participants had not seen advertising or promotional materials for KFPs. This suggests that a major component of increasing community members' engagement with their local shelters' KFPs may be increasing community members' awareness of programs through exposure to these advertisements. Furthermore, our results suggest that if local programs in Los Angeles County want to increase engagement among foster caregivers in predominantly Latinx, high-intake communities where they provide services, marketing to Spanish-language speakers should be a core component of these efforts given that Spanish language respondents were less likely to have encountered kitten fostering promotional materials.

This finding concerning differences in Spanish and English-language participants is not surprising given prior evidence that minority language speakers (and/or those not fluent in English) often experience linguistic isolation in the U.S. and Spanish-speaking Latinxs frequently experience reduced access to multiple forms of care and services (19). Moreover, a recent survey of 2,630 individuals disseminated by the Association for Animal Welfare Advancement (20) suggests that 95% of animal welfare workers in the U.S. report that English is their first language and only 6% are Hispanic/Latinx. Jenkins and Rudd (21) argue that disparate representation of minoritized population groups in animal welfare efforts is a barrier to the development of services, and approaches that center marginalized individuals and communities are needed to promote comprehensive strategies and drive culturally relevant approaches. Therefore, in addition to focusing on the development and dissemination of Spanish-language promotional materials, ensuring that the diversity of animal welfare workers mirrors that of the local community will likely be a critical component of increasing public engagement in cat management and fostering community-informed programs, particularly in the context of high-intake communities (22).

More than a quarter of the sample (i.e., 26%) were already engaged in fostering on their own without an animal shelter or rescue program. Accordingly, a majority of community members felt that the most common action taken for cats and kittens in their neighborhood was that they and/or other community members were taking care of them. It is also important to note that 19% and 20% of the sample felt like no one was taking care of cats and kittens in their neighborhood, respectively. In addition, only a small proportion of the sample (<6%) said they were brought to local shelters. Yet, more than two-thirds (69%) of participants who had already fostered cats and kittens on their own were open to fostering in partnership with an animal shelter and one-third of the *total* sample was open to fostering in partnership with a local animal shelter. Our finding that less than 20% of those interested in fostering had seen advertising for fostering programs suggests there is great opportunity to increase partnerships between residents of HKSI communities and local shelters and expand on the ongoing work being conducted by community members to care for community cats and kittens.

An interesting finding regarding participants' interest in fostering is that Spanish-language respondents were less likely than expected to be interested in fostering scenarios that did not involve the presence of the adult mother cat. Although more research is needed to understand this finding, a possible explanation is that those who already care for community cats and kittens, or know someone who has, may be more likely to be aware of the higher demand on individuals who foster kittens without an adult mother cat (e.g., bottle feeding). Although language and prior experience fostering were not statistically significantly associated in our sample (*p* = 0.09), the prevalence of prior fostering experience without an animal rescue organization was higher among Spanish-language respondents than those who completed the survey in English (33 vs. 28%, respectively). Thus, our Spanish-language respondents may have been more aware of the higher demand on individuals who foster without an adult mother cat (e.g., bottle feeding) and therefore be less likely than expected to be interested in this type of fostering scenario due to concerns about time and costs. Another potential explanation is that participants may be less likely to understand how to care for kittens without an adult mother due to a lack of access to Spanish-language resources on the topic or reduced access to digital technology.

To understand potential reasons why community members may be resistant or hesitant to engage in fostering, we examined 11 specific concerns about fostering and participants' corresponding level of worry. The most prevalent concerns in our sample were centered on having the time, money, and space to engage in fostering. Compared to the 2017 report by Maddie's fund, we found considerably higher levels of fostering-related concerns in our sample and relatively consistent rates of concern across each item (11). These differences may be explained by differences in sample diversity between the two studies and/or differences in our item phrasing and response scale. For example, the higher rate of worry in our sample may reflect the degree of situational vulnerability among participants in our sample. The report by Maddie's Fund did not provide sociodemographic data on the sample nor did it discuss how interest in and obstacles to fostering vary as a function of identity and/or resources; therefore, we can only speculate as to whether sociodemographic differences in the two samples may help to explain differences in rates of concerns between our samples. However, it is likely that our sample, representing a community with a high degree of situational vulnerability (e.g., lower than average income and education), experiences greater barriers to having and caring for animals and may have accompanying worries. These results suggest that another important step to increasing engagement in fostering programs is providing knowledge and resources that mitigate community members' concerns about fostering. Multilingual promotional and training materials for foster programs can help to provide community members with realistic estimates of the time and space requirements for fostering kittens, ensure that community members are aware that costs are covered by the organization (if applicable), and work with residents to identify alternative program strategies that can promote equitable opportunities for community members to engage in fostering and other animal welfare activities. Alternatively, animal welfare organizations could aim to identify what assets community members who are already taking care of these animals provide and adapt “typical” or common foster program requirements to utilize the expertise of those already engaged in caring for these community cats and kittens and supplement what is already being done.

Despite the concerns of prospective fosters, on average, those who were open to fostering could meet about six out of 10 of the common foster program requirements examined in the current study. Prevalent ways community members could regularly contribute included promoting adoptable kittens to friends and family (52%), giving plated food to kittens up to four times a day (45%), bottle-feeding kittens every 2–5 hours (36%), and hand feeding kittens up to 6 times a day (35%). Program requirements endorsed less frequently were keeping kittens in the home for 1 week to 1 month (29%) and those involving transportation. Our results suggest it may be beneficial to identify community members who are interested in fostering and work with them to create a tailored opportunity that builds on the program elements they are able to provide. Encouraging promotion of adoptable kittens to friends and family will be an important tool to keep altered kittens in the community, which can assist with promoting better management of cats and kittens in high shelter-intake areas. It may also help with the substantial concern of becoming too attached to the foster kittens by keeping their adoptive family close. In addition, if attending an online training session is the most prevalent barrier to participation, this suggests animal welfare organizations in these areas (or those with comparable levels of situational vulnerability to the neighborhoods examined in this study) should not rely solely on online, web-based trainings or fostering certifications and may need to offer multiple formats for these sessions. On the other hand, if, for example, housing restrictions (e.g., pet deposit, pet rent) are a primary barrier to keeping foster animals in the home for several weeks, shelters could consider and develop opportunities to partner with local landlords or housing associations to address this barrier to fostering and adoption. In our sample, transportation was a barrier for about one-third of the sample. For similar communities where residents experience more situational vulnerability, animal welfare organizations may benefit from working with community members to develop alternative sources of reliable and safe transportation for fosters that address this barrier or by providing in-home veterinary visits for foster animals and their caregivers.

Finally, results of this study suggest that the most effective approach for ensuring messaging about KFPs is impactful is to utilize promotional materials centered on animal welfare (i.e., kitten survival) and receiving support from the local animal welfare organization (e.g., covering all or some costs for the foster animal, 24/7 help line). It may be beneficial for future studies to examine how the source of information and message interact to influence perception and behavior. For example, we found that only 5% of our sample endorsed that a personal message from a current KFP volunteer would most encourage them to participate; however, this may differ based on whether the volunteer is an acquaintance, colleague, friend, or family member. Our findings also suggest that community members perceive text, email, social media, and mail communication among the top forms of communication for receiving information about foster programs. However, it is important to consider that social media may be less effective among Spanish-speaking community members and those over 55 years of age and may indicate reduced access to technology. These findings illustrate the importance of aligning communication and promotional materials with target populations and the role of multifaceted recruitment and marketing approaches in promoting diversity and equity among foster caregivers.

### Limitations

Although this study addresses an important gap and provides preliminary insights into how to engage community members in kitten fostering in HKSI areas, there are several limitations that warrant attention. First, this cross-sectional study utilized a small convenience sample of individuals who had already engaged with a large animal welfare organization to support animals. In addition, a majority were pet owners. We strategically targeted this group for the purposes of program development, hypothesizing that this group may be more willing to help animals in their community. However, rates of interest, awareness of cat and kitten overpopulation issues, and worries about fostering may not be reflective of HKSI and non-HKSI areas in LA County as a whole. Engaging community members who have had no contact or positive interactions with local animal welfare services may require different strategies than the ones proposed in this paper. We also recruited participants by text, meaning that we did not reach people without this technology and our findings regarding individuals' messaging preferences are likely biased. This is further complicated by the fact that only 56% of those who initiated the survey completed all survey questions and that we only surveyed a small percentage of those who were eligible, so bias in who self-selected to participate could be substantial.

Another notable limitation is that the current study did not examine whether the respondents lived in pet-friendly housing. Understanding whether interest in fostering is associated with respondents' living situations, and whether residential or housing policies permit pets, is critical to understand when assessing community members' capacity to foster. For example, a recent study found that communities of color and low-income communities in Texas were more likely than predominantly White and higher income communities to pay higher fees to keep companion animals in their homes (23). Understanding this potential barrier could help to identify whether those who are interested in fostering might be putting themselves at risk for fees or eviction due to a lack of compliance with housing restrictions, which has significant implications for the welfare of potential foster animals and their caregivers, as well as the sustainability of programs that aim to engage community members in high-intake areas. Relatedly, due to our small sample size, we examined Hispanic/Latinx identity as a “catch all” for all individuals who identified as Hispanic and/or Latino/a/x. However, there is great variability in culture and lived experiences across Latinx subgroups (e.g., Afrolatinidad and other multi-ethnic/racial experiences; Guatemalan vs. Mexican) and these differences cannot be meaningfully captured with our measurement approach. Similarly, the identities grouped in the “non-Hispanic/Latinx” group are diverse, and this categorization does not capture the complexity of the represented identities. Although a notable strength of our study is the strong representation of individuals from minoritized groups who have been underrepresented in animal welfare and human-animal interactions research, we acknowledge that our demographic groupings of “Latinx vs. non-Latinx” and “Spanish-language participants vs. English-language participants” is overly simplistic and cannot adequately capture the diverse ethnic, racial, and cultural experiences of these community members. Similarly, we also combined gender minority participants in a category with those who identified as women. As recently argued by Jenkins and Rudd (21), drawing from the work of Crenshaw (24), such methods are problematic and may lead to an erasure of complex identity-based experiences.

### Implications and Recommendations for Fostering Programs and Future Research

In addition to understanding the ways that community members can engage in fostering, and the barriers they experience to caring for community kittens, it is important that animal welfare organizations consider how individuals' identity and socio-contextual resources may impact their engagement in animal welfare initiatives. Collecting and examining sociodemographic data can help shelters understand how interest in and obstacles to fostering (and involvement in other animal welfare programming) may vary as a function of identity and/or resources. This information can ensure that foster programs adapt their strategies to equitably engage underrepresented communities in foster caregiving, which is particularly important in high-intake areas (9). It is well-known that rates of pet ownership vary across population groups, with White households having significantly higher rates of cohabitation with companion animals (all pets: 70%; cats: 33%) than Latinx (all pets: 60%; cats: 13%) and Black (all pets: 29%; cats = 6%) households (25–27). Moreover, prior research suggests that animal welfare volunteers are predominantly White (non-Hispanic), female, and have an income between USD $50,000 and $99,999 (28). It is in the best interest of animal welfare organizations to consider the implications of the rapidly changing U.S. population and prioritize efforts to diversify staff and identify opportunities for engaging ethnically and racially diverse, underrepresented, and/or situationally vulnerable individuals and communities in inclusive and welcoming animal welfare initiatives such as KFPs.

Due to the predominance of Hispanic/Latinx participants in the current study, we focus our implications section on this segment of the population. The Hispanic/Latinx community accounted for 52% of the U.S. population growth in 2019 and is the fastest growing in the U.S (15, 29). By 2045, this population group is projected to reach up to 24.6% of the U.S. population (30, 31). It is important that animal welfare and rescue stakeholders are mindful of distrust and avoidance of health services among Latinx immigrants, which may extend to animal health and welfare programs and services (32). Testing whether specific advertisement, proactive recruitment and/or community engagement approaches are associated with increases in fostering over time, as well as diversity among program participants, could help to identify low-cost, high-reward strategies to engage high-intake communities in existing fostering programs.

Community-informed strategies to engage and retain Latinx individuals (and other historically and currently minoritized, marginalized, and underrepresented groups) in fostering efforts could help to expand the capacity of KFPs, drive enhanced and culturally relevant models of animal care, and improve the sustainability of these animal welfare programs by leveraging community wisdom, cultural values (e.g., familismo^1^, collectivism^2^) and the expertise and lived experiences of individuals who have been systemically excluded. To achieve community- and culturally relevant models that are sustainable, it would be beneficial to employ participatory action research methodologies, which involve researchers and participants (in this case, community members of HKSI areas) working together as co-researchers, to drive programmatic changes (21). Moreover, qualitative methods may help to identify critical knowledge and recommendations from community members who have already been engaged in caring for community cats and kittens. To this end, it may be important for animal welfare organizations to reframe how they approach engaging individuals in fostering. Instead of asking *how can we increase community members' participation in our programs*, it may be more important to understand *how animal welfare organizations can support community members in the efforts they have already been successful in employing* to address care and management of community cats. Such efforts may require shelters to rethink the nature and structure of KFPs and redirect funds to achieve these initiatives. Of note, *Promatoras de salud*, or “promotoras” is a Spanish-language term for a culturally competent health care worker who provides or promotes culturally relevant health education, teaches wellness knowledge and behaviors, and assists individuals in navigating healthcare systems. *Promatora* models have been found to be highly successful in improving public health knowledge and behaviors in a variety of Latinx/Hispanic communities in the U.S. Prior research on the experiences of promotoras has identified veterinary medicine as an area where these workers feel their skills could be useful, but are not currently being utilized (35). Animal welfare organizations may benefit from considering similar models to promote companion animal health and welfare and address the care and management of community cats and kittens in high-intake Latinx communities.

## Conclusion

Our study identified that a notable proportion of residents of HKSI zip codes in Los Angeles County were already engaged in caring for cats and kittens in their neighborhood and had fostered kittens without the help of an animal welfare organization. We identified several opportunities and barriers to engaging community members from HKSI areas in fostering kittens in partnership with local shelters. Opportunities for increasing engagement included, but were not limited to, improving the promotion advertisements using specific messaging approaches (i.e., animal welfare- and cost-focused) as well as improving the utilization and dissemination of Spanish-language materials. Helping community members have realistic expectations of the time, resources, and support they will get from the animal welfare organization may improve engagement in KFPs, as well as identifying alternative resources and supports to assist community members in serving animals in their community (e.g., safe and reliable transportation, pet deposits, pet rent). Community-based participatory action research and qualitative methodologies may be particularly useful approaches to better understand the relationship between HKSI communities and animal welfare organizations. Furthermore, undertaking these methodologies to identify community wisdom and drive innovative adaptations or restructuring of KFPs may assist animal welfare organizations in developing better and more equitable partnerships that are culturally informed and sustainable in local communities.

## Data Availability Statement

The data are available upon request by contacting ASPCAResearch@aspca.org.

## Ethics Statement

This study was approved by Advarra IRB (Protocol Number: Pro00050534). Human participants provided informed consent to participate in the study.

## Author Contributions

SM, GM, TF, and DO: conceptualization. SM, GM, TF, DO, and AM: methodology, review, and editing. SM and GM: analysis. SM, GM, and AM: writing. All authors contributed to the article and approved the submitted version.

## Funding

This study was funded and performed by the ASPCA.

## Conflict of Interest

The authors declare that the research was conducted in the absence of any commercial or financial relationships that could be construed as a potential conflict of interest.

## Publisher's Note

All claims expressed in this article are solely those of the authors and do not necessarily represent those of their affiliated organizations, or those of the publisher, the editors and the reviewers. Any product that may be evaluated in this article, or claim that may be made by its manufacturer, is not guaranteed or endorsed by the publisher.

## Footnotes

^1^Familismo is a cultural value that describes an orientation to and a connectedness with one's family and is rooted in collectivistic ideology (33).

^2^Collectivism is a social and cultural orientation that emphasizes and prioritizes the interdependence of a group and often values close, supportive, and nurturing interpersonal relationships over individualistic behaviors and attitudes (34).
